# Complete genome sequence of *Allohahella* sp. A8 isolated from the east Gakkel Ridge sediment

**DOI:** 10.1128/mra.00638-26

**Published:** 2026-09-21

**Authors:** Ge-Yi Fu, Yong-Lian Ye, Lin Xu, Cong Sun, Xue-Wei Xu

**Affiliations:** 1 Key Laboratory of Marine Ecosystem Dynamics, Ministry of Natural Resources and Second Institute of Oceanography, Ministry of Natural Resources, Hangzhou, People's Republic of China; 2 College of Life Sciences and Medicine, Zhejiang Sci-Tech University, Hangzhou, People's Republic of China; 3 Shaoxing Biomedical Research Institute of Zhejiang Sci-Tech University Co., Ltd., Zhejiang Engineering Research Center for the Development Technology of Medicinal and Edible Homologous Health Food, Shaoxing, People's Republic of China; 4 National Deep Sea Center, Ministry of Natural Resources, Qingdao, People's Republic of China; Montana State University, Bozeman, Montana, USA

**Keywords:** complete genome, *Allohahella*, hydrothermal sediment

## Abstract

We report the complete genome sequence of *Allohahella* sp. A8 isolated from one deep-sea hydrothermal sediment sample collected in the east Gakkel Ridge rift zone, Arctic Ocean. The genome consists of a circular chromosome (4,665,952 bp; GC content 56.0%), containing 4,103 coding genes and 54 RNA genes.

## ANNOUNCEMENT

The genus *Allohahella*, belonging to the class *Gammaproteobacteria*, the order *Oceanospirillales*, and the family *Marinobacteraceae*, is Gram-negative, aerobic or facultatively anaerobic, and morphologically characterized as irregularly rod and the absence of flagella (1, 2). To date, two *Allohahella* species have been validly identified, and both were isolated from marine environments, including coastal seawater in western Antarctica (3) and seawater of an amphioxus breeding zone (2). These discoveries of *Allohahella marinimesophila* and *Allohahella antarctica* indicate that strains belonging to the genus *Allohahella* have the potential to survive across a wide range of marine habitats. This is the first species of the genus *Allohahella* isolated from hydrothermal environments, providing new insights into its adaptation and ecological roles in Arctic hydrothermal habitats.

Strain A8 was isolated from deep-sea sediment core collected by gravity corer in the eastern Gakkel Ridge rift zone, Arctic Ocean, at a depth of 4,475 m (85.09° N, 95.85° E) during China’s 12th Arctic Ocean scientific expedition onboard R/V *Xuelong 2*. The Gakkel Ridge spans the ice-covered Arctic Ocean with a length of 1,800 km from Greenland to the Russian Shelf and is characterized as an ultraslow spreading mid-ocean ridge, forming a distinct spreading mode and hydrothermal environment (4, 5). Geological explorations confirm that numerous hydrothermal systems exist along the Gakkel Ridge, covering over 75% of this ridge (6, 7). After rinsing with sterile 3.0% NaCl (wt/vol) and homogenized, the sample was spread on modified Marine Agar 2216 supplemented with 1.0 g/L peptone and 0.1 g/L yeast extract. Following incubation at 28°C, a white-colored colony was selected and designated as strain A8. Strain A8 was deposited into the Marine Culture Collection of China (MCCC 1K10341).

After cells were grown in Marine Broth 2216 (BD Difco, USA; MB) at 28°C for 4 days, cells were harvested by centrifugation and sent to Guangdong Magigene Biotechnology Co., Ltd. (Guangzhou, PR China) for genomic DNA extractions and sequencing. DNA extractions were carried out by using a modified SDS-based DNA extraction method (8). Extracted genomic DNA was qualitatively checked via agarose gel electrophoresis and quantified with a Qubit 4.0 Fluorometer (Thermo Fisher Scientific, USA) prior to library construction. Genomic sequencing was conducted using PacBio Sequel II (Pacific Biosciences, USA) and Illumina NovaSeq PE150 (Illumina, Inc., USA).

Default parameters were used unless specified. Illumina reads were filtered with Trimmomatic v0.39. PacBio HiFi reads were directly produced via the platform’s SMRTLink toolkit; these sequences had an N50 > 6,000 bp and passed built-in quality filters, requiring no extra trimming or length filtering. Clean Illumina and HiFi reads were co-assembled with hifiasm v0.19.6, followed by two rounds of Pilon v1.24 polishing using Illumina reads mapped with bwa-mem2 v2.2.1. Circular topology of the chromosome was verified by Circlator v1.5.5. Genome completeness and assembly statistics were assessed by BUSCO v5.7.1 (bacteria_odb10 database) and QUAST v5.3.0, respectively. PacBio generated 113,547 filtered CCS HiFi reads totaling 799,091,241 bp, with an N50 of 7,401 bp and average read length of 7,037.54 bp, yielding ~171.26× theoretical sequencing depth.

**Fig 1:**
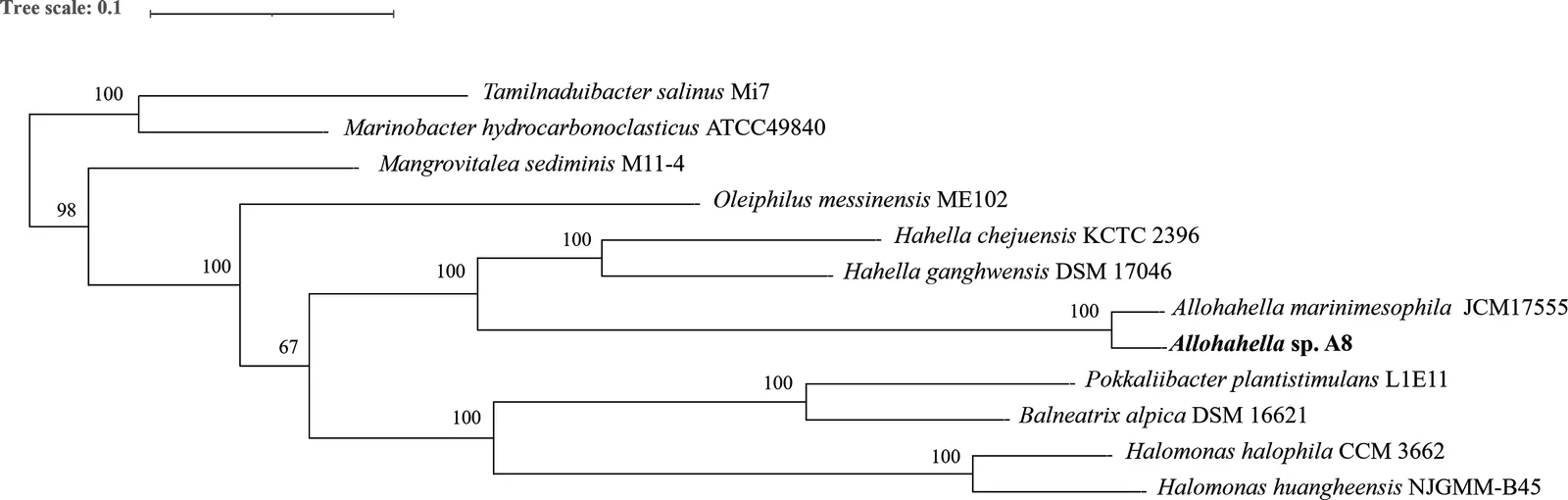
Maximum-likelihood phylogenomic tree of the genus *Allohahella* based on 120 bacterial marker genes. Bar, 0.1 per amino acid substitutions.

Gene prediction was performed using Prokka v1.11 (9). The 16S ribosomal RNA (rRNA) gene sequence identity was analyzed against the EzBioCloud webserver (10). Phylogenomic reconstruction was performed using IQ-Tree v1.6.12 based on 120 bacterial marker genes from GTDB-Tk v2.3.2 (11). The genome of strain A8 contains a single circular chromosome of 4,665,952 bp, with a GC content of 56.0%. Annotation was conducted using the National Center for Biotechnology Information (NCBI PGAP v6.10) with default parameters. A total of 4,103 protein-coding genes, 41 transfer RNA (tRNA) genes, 9 rRNA genes, and 4 non-coding (ncRNA) genes were identified (Table 1). Comparative analysis of the 16S rRNA gene sequences showed that strain A8 shared the sequence identities of 98.9%, 96.6%, and 93.7% with *Allohahella marinimesophila* H94^T^, *A. antarctica* IMCC3113^T^, and *Hahella chejuensis* KCTC 2396^T^. A maximum-likelihood phylogenetic tree based on 120 bacterial marker genes showed that *Allohahella* sp. A8 formed a stable, independent clade with A. *marinimesophila* JCM17555 and clustered within the clade with genus *Hahella* (Fig. 1).

**TABLE 1:** General statistics of *Allohahella* sp. A8

Attribute	Value
Size (bp)	4,665,952 bp
Genome topology	Circular
Contigs	1
G + C content (%)	56.0
No. of chromosome	1
No. of plasmids	0
No. of predicted coding sequences (CDSs)	4,103
tRNAs	41
rRNAs(5S/16S/23S)	9 (3/3/3)
ncRNAs	4

## Data Availability

The complete genome sequence of strain *Allohahella* sp. A8 has been deposited in NCBI under accession number CP172312 with BioProject and BioSample accession numbers PRJNA1102269 and SAMN41015456, respectively. The raw data for the genome sequence of strain A8 have been deposited in NCBI Sequence Read Archive database under the accession number SRR38172290.
